# A Comprehensive Analysis In Silico of *KCS* Genes in Maize Revealed Their Potential Role in Response to Abiotic Stress

**DOI:** 10.3390/plants13243507

**Published:** 2024-12-16

**Authors:** Xinyi Chen, Aixia Zhang, Chenyan Liu, Muhammad Saeed, Junyi Li, Ying Wu, Yunhao Wu, Haijing Gu, Jinchao Yuan, Baohua Wang, Ping Li, Hui Fang

**Affiliations:** 1 Scientific Observing and Experimental Station of Maize in Plain Area of Southern Region, Ministry of Agriculture and Rural Affairs, School of Life Sciences, Nantong University, Nantong 226019, China; 15062480996@163.com (X.C.); a17662451703@163.com (A.Z.); 15358302605@163.com (J.L.); 15312928136@163.com (Y.W.); wuyunhao0323@126.com (Y.W.); 15936135237@163.com (H.G.); bhwang@ntu.edu.cn (B.W.); 2 Department of Agricultural Sciences, Government College University, Faisalabad 38000, Pakistan; saeed_pbg@gcuf.edu.pk; 3 Qidong Ruichao Farm, Nantong 226200, China

**Keywords:** *Zea mays*, *KCS* genes, gene family, abiotic stress response

## Abstract

β-ketoacyl-CoA synthase (KCS) enzymes play a pivotal role in plants by catalyzing the first step of very long-chain fatty acid (VLCFA) biosynthesis. This process is crucial for plant development and stress responses. However, the understanding of *KCS* genes in maize remains limited. In this study, we present a comprehensive analysis of *ZmKCS* genes, identifying 29 *KCS* genes that are unevenly distributed across nine maize chromosomes through bioinformatics approaches. These ZmKCS proteins varied in length and molecular weight, suggesting functional diversity. Phylogenetic analysis categorized 182 KCS proteins from seven species into six subgroups, with maize showing a closer evolutionary relationship to other monocots. Collinearity analysis revealed 102 gene pairs between maize and three other monocots, whereas only five gene pairs were identified between maize and three dicots, underscoring the evolutionary divergence of *KCS* genes between monocotyledonous and dicotyledonous plants. Structural analysis revealed that 20 out of 29 *ZmKCS* genes are intronless. Subcellular localization prediction and experimental validation suggest that most ZmKCS proteins are likely localized at the plasma membrane, with some also present in mitochondria and chloroplasts. Analysis of the *cis*-acting elements within the *ZmKCS* promoters suggested their potential involvement in abiotic stress responses. Notably, expression analysis under abiotic stresses highlighted *ZmKCS17* as a potential key gene in the stress response of maize, which presented an over 10-fold decrease in expression under salt and drought stresses within 48 h. This study provides a fundamental understanding of *ZmKCS* genes, paving the way for further functional characterization and their potential application in maize breeding for enhanced stress tolerance.

## 1. Introduction

Very long-chain fatty acids (VLCFAs) in plants play pivotal roles in lipid metabolism, development, and response to abiotic stresses. They contribute to the structural integrity and functional adaptability of cellular membranes, which are crucial for plant survival under various stress conditions [1,2,3,4]. The synthesis of VLCFAs in higher plants is a complex process that involves the elongation of C16 and C18 precursors into fatty acids with C22 or more carbon atoms [5,6]. This biosynthesis occurs in plastids, with further elongation occurring in the endoplasmic reticulum [5,6]. VLCFAs are fundamental for maintaining cellular membrane structure and functionality, particularly in resistance to biological and abiotic stimuli, and offer protection against pathogens, salinity, drought, and extreme temperature [3,4,7,8]. These stress factors prompt plants to biosynthesize and accumulate VLCFAs in various tissues, participating in cellular processes such as nuclear pore formation, cell signaling, hormone regulation, and lipid metabolism [9,10,11,12]. Notably, plant cuticular waxes, which are primarily composed of VLCFAs, resist various environmental stresses by establishing a hydrophobic barrier that reduces nonstomatal water loss and provides protection against a range of biotic and abiotic stresses [13]. Thus, VLCFAs possess significant biological functions and application value.

Ketoacyl-CoA Synthase (KCS) genes are essential in plants and encode enzymes that catalyze the critical elongation step in VLCFA synthesis. These genes determine the rate of VLCFA biosynthesis and the length of carbon chains, linking them to diverse physiological and developmental pathways such as plant wax formation, pollen fitness, and stress responses. The functions of *KCS* genes have been extensively studied in many plants, with numerous *KCS* members identified across various plants: 25 in sorghum [4], 31 in *Allium fistulosum* [14], 31 in soybean [15], 32 in *Brassica* [16], 33 in barley [17], 32 in passion fruit [18], 30 in *Carya illinoinensis* [19], 20 in yellow horn [20], 30 in peanut [21] and 35 in pear [22], among others. Advances in genetic manipulation have facilitated detailed functional analyses of *KCS* genes, revealing their significant impact on the regulation of various plant biological processes beyond VLCFA biosynthesis [23,24,25,26,27,28,29]. For instance, in *Arabidopsis thaliana*, *KCS1*, *KCS3*, *KCS12*, and *KCS19*, which encode fatty acid 3-ketoacyl-CoA synthases, have been shown to regulate wax biosynthesis and maintain cuticle integrity [23,24,25]. Similarly, in Chinese cabbage, *BrWAX3* also plays a role in epidermal wax synthesis [26], whereas *KCS4* is involved in root and pollen tube growth in *Arabidopsis* [27]. A seed coat-specific β-ketoacyl-CoA synthase, *KCS12*, is critical for preserving seed physical dormancy in *Medicago truncatula* [28], and another 3-ketoacyl-CoA synthase, WFL, participates in the late stages of leaf and flower development in the same species [29]. Emerging studies are exploring the role of *KCS* genes in enhancing crop resilience to biotic and abiotic stresses, opening new avenues in agricultural biotechnology [30]. By regulating VLCFAs and waxy biosynthesis, plant *KCS* genes can effectively cope with biological and abiotic stresses. The overexpression of the *VvKCS12* and *VvKCS14* genes from grape, the *MsKCS10* from alfalfa, and the *AtKCS19* genes in *Arabidopsis* have been shown to enhance drought tolerance of transgenic plants [24,31,32]. The overexpression of *chenopodium quinoa CqKCS2B*.1 and *Arabidopsis AtKCS19* genes have been shown to increase salt tolerance [24,33]. In cotton, *GhKCS13* modulates lipid and oxylipin biosynthesis to regulate the cold response [34], and *GaKCS6* from *Gossypium arboretum* plays a role in tolerance against whitefly [35].

Maize, a globally significant food crop, plays an irreplaceable role in global food security and nutrition [36]. Abiotic stresses pose a significant threat to maize, severely affecting its yield and global food security [37]. While numerous studies have highlighted the importance of *KCS* genes in helping plants cope with stresses, and *KCS* genes have been investigated in several plants, the role of *KCS* genes in maize remains largely unexplored. Herein, we report a compressive analysis of the *KCS* gene family in maize, including phylogenetic evolution, gene structure, *cis*-acting elements, and expression profiles in different tissues or under stress treatments. These findings will aid in further studies of the biological functions of *ZmKCSs* in development and stress responses in maize.

## 2. Results

### 2.1. Identification and Chromosomal Location Analysis of KCS Genes in Maize

**Figure 1:**
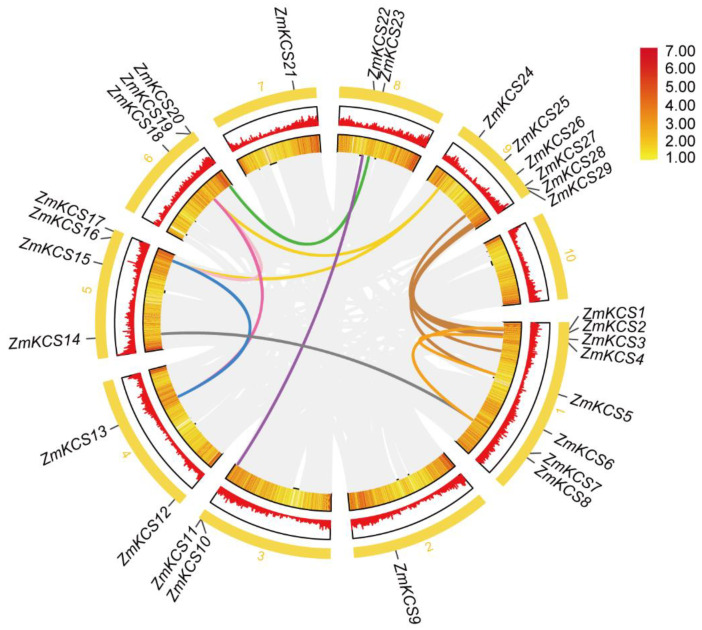
Chromosomal distribution of maize *KCS* genes and their interchromosomal relationships. The innermost ring represents the syntenic blocks across the maize B73 genome, with grey lines indicating all such blocks. The colorful lines denote the collinear blocks of *ZmKCS* genes within the maize genome. The subsequent yellow and red rings correspond to gene density and the GC ratio, respectively, with each ring reflecting these genomic features at their respective locations. The outermost ring shows the physical positions of the *ZmKCS* genes within the maize genome.

After filtration through the SMART, NCBI-CDD, and Pfam databases, 29 *KCS* genes within the maize B73 genome were identified and designated as *ZmKCS1*-*ZmKCS29* according to their physical positions on the maize chromosomes (Figure 1; Figure S1). A chromosome map was generated to investigate the genomic distribution of the *ZmKCS* genes. The results revealed that the 29 *ZmKCS* genes are unevenly distributed across the nine maize chromosomes except chromosome 10, with most genes located at both ends of each chromosome. The number of genes on each chromosome varies from 1 to 8. Chromosome 1 contains the greatest number of *ZmKCS* genes, while chromosomes 2 and 7 each have only one *ZmKCS* gene. (Figure 1; Figure S1). The 29 ZmKCS proteins exhibit varying sequence lengths, ranging from 222 amino acids (ZmKCS29) to 547 amino acids (ZmKCS4), with molecular weights ranging from 24,360.9 Da (ZmKCS29) to 60,645.4 Da (ZmKCS4) (Table S1), indicating a positive correlation between their molecular weight and length. These findings suggest that there may be differences in the domains, expression sites, expression patterns, and tissue specificity of these *KCS* genes, which ultimately lead to functional diversity. Furthermore, the theoretical isoelectric point (PI) of all the ZmKCS proteins was greater than 7, ranging from 7.2 (ZmKCS9) to 10.3 (ZmKCS29) (Table S1), indicating the alkaline nature of the maize KCS family proteins.

To understand the evolutionary relationships within the *KCS* gene family, we analyzed the gene duplication events in the maize *KCS* gene family. Gene duplication is one of the primary drivers of gene family expansion and can originate from whole-genome duplications, segmental duplications, tandem duplications, and transposition events. Specifically, in our study, we identified a total of 15 gene pairs involving 18 *KCS* genes that exhibit duplication relationships (Figure 1), which indicates that gene replication plays an important role in the evolution of the maize *KCS* gene family. These gene pairs likely originated from ancient genomic duplication events that played crucial roles in the evolutionary history of the maize genome.

### 2.2. Evolutionary Analysis of ZmKCS Proteins

**Figure 2:**
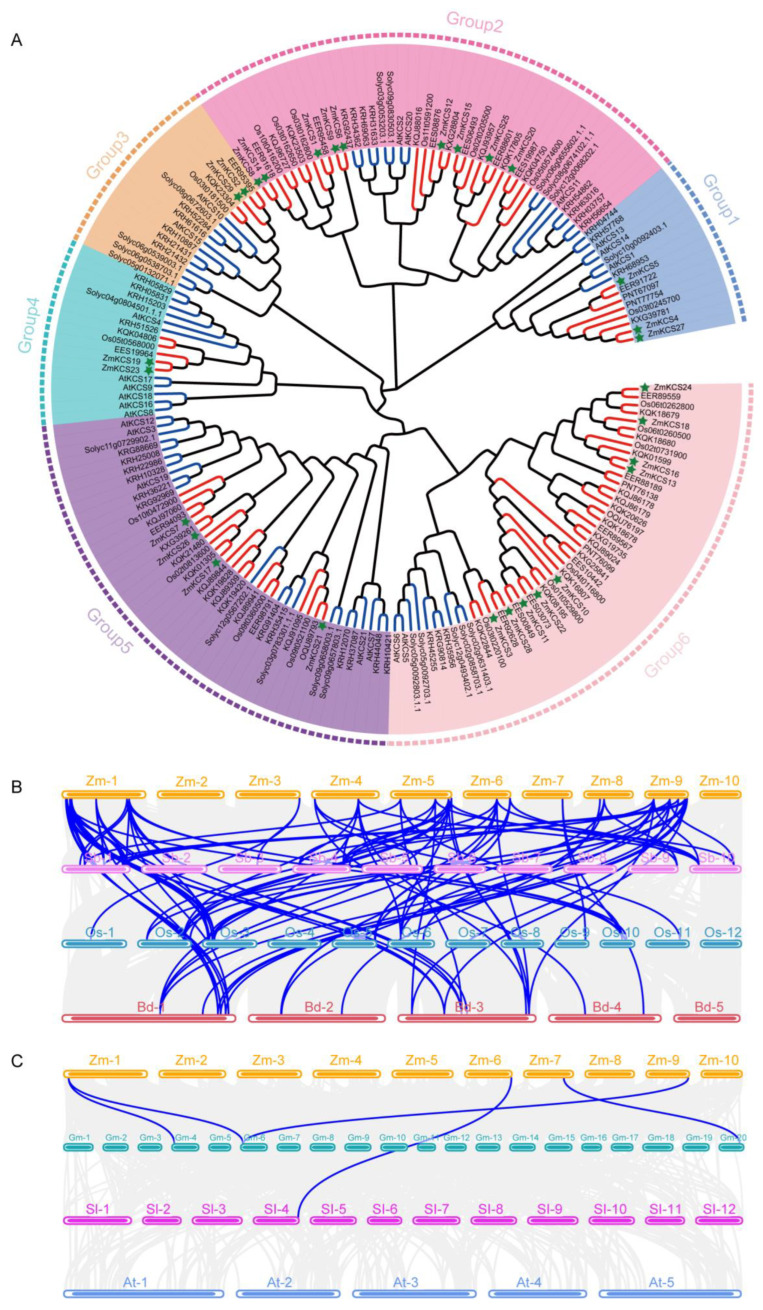
Phylogenetic analysis of *KCS* gene family across different species. (**A**) This section presents a phylogenetic tree of *KCS* gene family members from seven species, including maize and three monocots (sorghum, rice, *Brachypodium distachyon*), as well as three dicots (*Arabidopsis thaliana*, tomato, soybean). The tree was constructed using MEGA 7.0 software employing the maximum likelihood (ML) method. The tree is organized into six subgroups, each identified by a distinct background color. Monocot branches are highlighted in red, with maize *KCS* genes marked by green stars. The abbreviations used are as follows: EER, EES for sorghum; KQJ, KQK, PNT for *Brachypodium distachyon*; At for *Arabidopsis thaliana*; Zm for maize; Os for rice; solyc for tomato; and KRH for soybean. (**B**) This panel shows the results of a collinearity analysis of *KCS* genes between maize and three dicots: *Glycine max* (Gm), *Solanum lycopersicum* (Sl), and *Arabidopsis thaliana* (At). (**C**) This panel shows the collinearity analysis of *KCS* genes between maize and three monocots: *Sorghum bicolor* (Sb), *Oryza sativa* (Os), and *Brachypodium distachyon* (Bd). The background gray lines represent genome-wide collinear blocks, while the blue lines specifically highlight the collinearity of *KCS* genes, illustrating evolutionary connections and genomic conservation across these species.

To elucidate the evolutionary relationships among KCS proteins across both monocot and dicot plants and establish a comprehensive classification system, we constructed a phylogenetic tree using the full-length sequences of KCS proteins from seven species, including four monocotyledonous plants, namely, maize (29 genes), *sorghum bicolor* (25 genes), rice (19 genes), and *Brachypodium distachyon* (32 genes), as well as three dicot species, namely, *Arabidopsis thaliana* (21 genes), soybean (35 genes), and tomato (21 genes). Our objective was to provide a detailed evolutionary framework that highlights the diversification and conservation of KCS proteins within these plant species. A total of 182 KCS proteins were categorized into six subfamilies, designated as Group 1–Group 6, with each group containing 15 (Group 1)–48 (Group 6) *KCS* members (Figure 2A). The ZmKCS proteins were distributed across all six subgroups, with Group 2 and Group 6 hosting the greatest number of ZmKCS proteins (9 in each). In contrast, Group 3 and Group 4 had the fewest ZmKCS proteins, each with only two, whereas Group 1 and Group 5 contained three and four ZmKCS proteins, respectively. Moreover, phylogenetic analysis revealed that maize KCS proteins are more closely related to the evolutionary branches of monocot plants, indicating sequence differences and potential functional differentiation in *KCS* genes between monocots and dicots. Furthermore, the closer branching of the maize and sorghum KCS proteins within each subgroup suggests a closer evolutionary relationship between the maize and sorghum *KCS* genes, followed by the maize and rice genes.

In addition, we conducted a collinearity analysis to explore the evolutionary relationships of *KCS* genes among maize and six other species. Our findings indicate a significant divergence in *KCS* genes between monocotyledonous and dicotyledonous plants. Among the monocotyledons, the greatest number of collinear gene pairs was observed between maize and *Brachypodium distachyon* (36), followed by sorghum (35) and rice (31) (Figure 2B). In stark contrast, the number of collinear gene pairs between maize and the three dicotyledonous plants—soybean, tomato, and *Arabidopsis thaliana*—was minimal, with only 4, 1, and 0 pairs identified, respectively (Figure 2C). These results underscore the substantial evolutionary divergence of *KCS* genes between monocots and dicots. On the basis of this divergence, we hypothesize that the *ZmKCS* genes may have evolved from homologous genes in other monocots, sharing a common ancestry with sorghum and rice that can be traced back to ancient grass species.

### 2.3. Gene Structure and Conserved Motif Analysis of ZmKCS Genes

**Figure 3:**
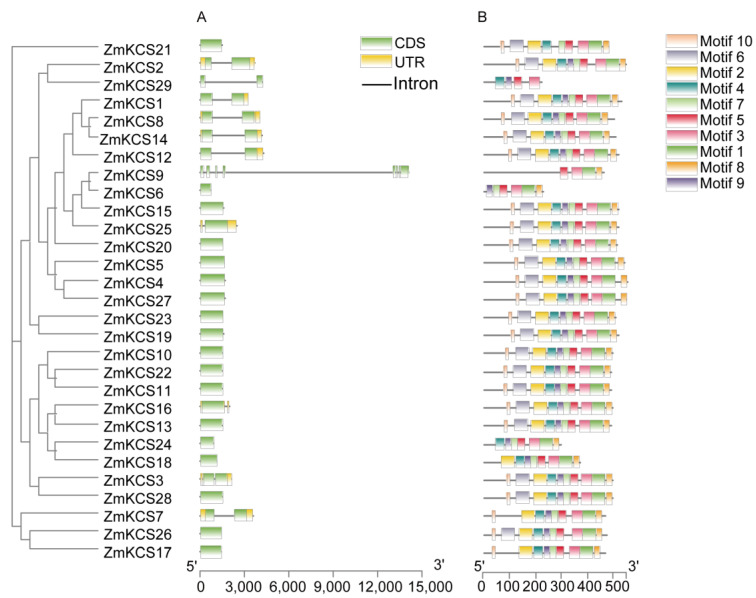
Gene structure and conserved motifs in *ZmKCS* genes. (**A**) Exon–intron structure of *ZmKCS* genes. (**B**) Motif analysis of ZmKCS proteins. Ten conserved motifs across 29 ZmKCS proteins, with each conserved motif represented by a unique color. Motif lengths are proportional to their representation in each protein.

To elucidate the structural composition of each *ZmKCS* gene, we compared the cDNA and DNA sequences of each *ZmKCS* gene to deduce their exon–intron structures (Figure 3A). The results revealed that the majority of the *ZmKCS* genes presented relatively simple structures, with the number of introns ranging from 0 to 7 across the 29 *ZmKCS* genes. A significant proportion of these genes (20 genes, ~69.0%) have open reading frames that are intronless, whereas eight *ZmKCS* genes (~28.0%) contain a single intron. The 20 intronless genes were distributed across all subgroups except for Group 3, and those with a single intron are found in all subgroups except for Group 1. Notably, genes in Group 1 and Group 4 are entirely intronless, whereas those in Group 3 have only one intron. Furthermore, *ZmKCS9* in Group 2 has a more complex structure, featuring multiple exons and introns. These observations suggest that genes within the same subgroup tend to share similar exon–intron structures with conserved intron positions, indicating a close evolutionary relationship within the *KCS* gene family.

To further identify the characteristic regions of the ZmKCS proteins, we employed MEME v5.5.5 software to predict 10 conserved motifs within the ZmKCS protein sequences (Figure 3B). The distribution of these motifs was notably consistent, with all the ZmKCS protein sequences containing conserved motifs 3 and 5. These findings suggest that these two conserved motifs may play crucial roles in the functional integrity of the maize *KCS* genes. In addition, most ZmKCS proteins have conserved motifs 1, 4, 7, 8, and 9. However, ZmKCS6, ZmKCS9, ZmKCS24, and ZmKCS29 lack conserved motifs 2, 6, and 10. The variation in the distribution of conserved motifs may imply that these genes have evolved distinct functions within the maize *KCS* gene family.

### 2.4. Multiple Sequence Alignment, Secondary Structure Prediction, and Three-Dimensional Modeling of the ZmKCS Proteins

To explore the functional domain of the *ZmKCS* genes, we performed multiple sequence alignments via ClustalW within MEGA 7.0 software. This alignment included the 29 identified ZmKCS and 21 AtKCS protein sequences. The analysis revealed two significant conserved structural domains: the FAE1_CUT1_RppA domain and the ACP_syn_III_C domain (Figure S2). The sequences within these domains exhibited a high degree of conservation, underscoring their essential role in the functionality of maize *KCS* genes. Moreover, a notable level of conservation was observed in the C-terminal and central regions of these proteins, whereas the N-terminal region presented relatively lower conservation.

Given the close relationship between protein structure and function, we predicted their secondary structures and constructed three-dimensional models for all ZmKCS proteins to deepen our understanding of the function of ZmKCS proteins. Their secondary structure was found to be primarily composed of α-helices, extended strands, β-turns, and random coils (Table S2). α-helices were the most prevalent, constituting 40.20% to 49.60% of the secondary structure, followed by random coils (32.29% to 39.22%), extended strands (11.29% to 18.02%), and β-turns (4.52% to 7.07%).

The three-dimensional structures of the ZmKCS proteins were modeled via homology modeling (Figure S3). These models revealed that ZmKCS proteins share similar three-dimensional conformations, facilitating a clear observation of the composition and positions of secondary structures within ZmKCS proteins. The secondary structure elements, α-helices, random coils, extended strands, and β-turns, further fold into a compact globular spatial structure through interactions between side-chain groups and the maintenance of various secondary bonds. This structural stability is crucial for the biological activity of proteins.

### 2.5. Analysis of the Subcellular Localization of the ZmKCS Proteins

**Figure 4:**
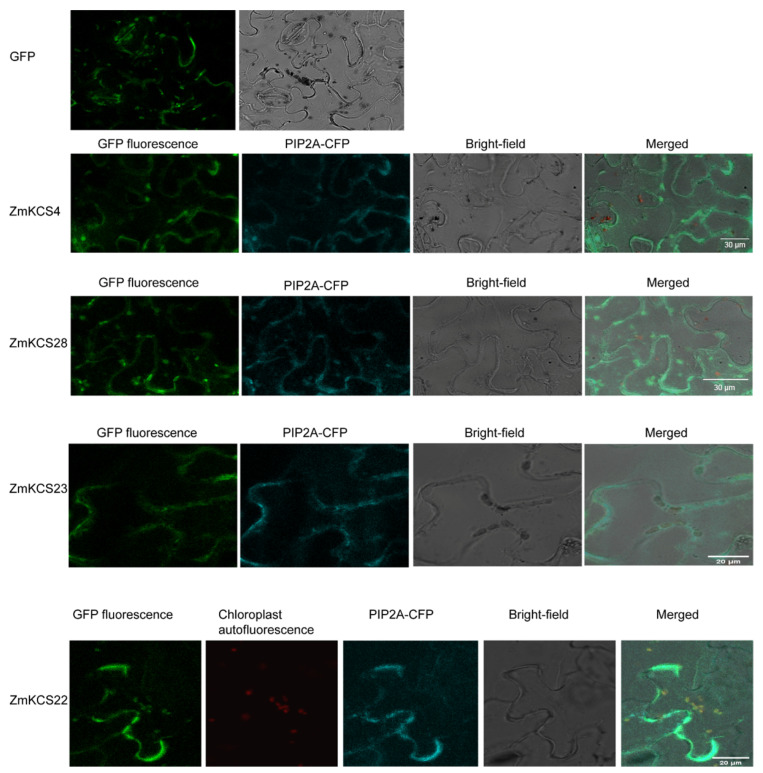
Subcellular localization of four of the *ZmKCS* genes in tobacco epidermal cells. The KCS-GFP fusion proteins are predominantly localized to the cell membrane and chloroplasts, as indicated by their fluorescence, indicating specific localization patterns.

Exploring the expression sites of proteins can provide clues for the study of protein function and protein interactions. Thus, the expression sites of ZmKCS proteins in the cell were predicted (Figure S4). The analysis indicated that a majority of ZmKCS proteins are likely to be localized at the plasma membrane, with 20 proteins accounting for approximately 69% of the total. The mitochondria were predicted to contain seven proteins (~24%), and chloroplasts were estimated to contain two proteins (~7%) (Figure S4). To substantiate these predictions, experimental validation was conducted on four randomly selected ZmKCS proteins. Fusion proteins of ZmKCS with GFP were constructed and introduced into tobacco leaves for expression, and confocal microscopy was used to observe the expression sites. By fusing GFP with the cyan membrane marker PIP 2A-CFP, it was determined that ZmKCS4, ZmKCS23, and ZmKCS28 are localized to the cell membrane. Furthermore, ZmKCS22 displayed GFP signals in both tobacco chloroplasts and the cell membrane, which correlated with the red chloroplast autofluorescence signal and the cyan PIP 2A-CFP signal in tobacco epidermal cells (Figure 4). These findings confirm that the ZmKCS22 protein is localized to both the cell membrane and chloroplasts, which is in accordance with the initial prediction outcomes.

### 2.6. Cis-Acting Element Analysis of the ZmKCS Gene Promoter

To delve into the potential regulatory roles of *ZmKCS* genes in various physiological processes and in response to stresses, we isolated the 1.5 kb promoter sequences of these genes and analyzed their potential *cis*-acting regulatory elements (CAREs) via the PlantCARE online tool. Our analysis identified a total of 21 CAREs within the promoter regions of *ZmKCS* genes that are associated with the abiotic stress response and hormone signaling (Figure S5). These elements included six stress-responsive elements, including MBS, LTR, TC-rich, CCAAT-box, W box, and WUN-motif, as well as 15 hormone-responsive elements such as ABRE, ARE, AuxRR-core, CGTCA-motif, ERE, G-box, GARE-motif, I-box, MYB, STRE, RY-element, TCT-motif, TGA-element, TGACG-motif, and TCA-element.

The presence of ABREs, associated with the abscisic acid response, was detected in 26 *ZmKCS* genes. The CGTCA-motif and TGACG-motif, both of which are involved in the jasmonic acid response, were found in 22 *ZmKCS* genes. AuxRR-core, related to the auxin response and RY-elements, implicated in seed-specific regulation, were identified in four *ZmKCS* genes. W-boxes, which are involved in wounding and pathogen response, and LTRs, which are associated with the low-temperature response, were detected in 12 *ZmKCS* genes. Additionally, STRE and TCA-elements, both linked to the salicylic acid response; TGA-elements, related to the auxin response; GARE-motifs, involved in the gibberellin response; ARE, associated with anaerobic induction; TC-rich elements, implicated in the defense and stress response; MBS, linked to drought induction; CCAAT box, related to the high-temperature response; and WUN motif, involved in wounding induction, were present in 25, 7, 15, 6, 19, 5, 20, 21, and 9 *ZmKCS* genes, respectively. Furthermore, the G-box, TCT-motif, Box 4, and I-boxes, which are related to the light response, were identified in 27, 13, 11, and 5 *ZmKCS* genes, respectively. Notably, the MYB *cis*-acting element, which plays a role in controlling plant secondary metabolism, regulating cell morphogenesis, and responding to environmental factors, was found in all the *ZmKCS* genes. These findings strongly suggest that *KCS* genes in maize may play a role in mediating responses to abiotic or biotic stresses as well as in hormone signaling pathways.

### 2.7. Expression Profiling and Pattern Analysis of ZmKCS Genes Under Abiotic Stresses

To further investigate the potential functions of the *ZmKCS* genes in maize growth and development, as well as their response to abiotic stresses, we carried out a comprehensive analysis of the transcriptome data from 12 distinct tissues for 25 *ZmKCS* genes, as reported in a previous study [38]. Our detailed examination revealed diverse expression patterns among the maize *KCS* genes. A majority of these genes were expressed at low levels or were nearly absent in embryos, endosperms, and seeds. In contrast, these genes were more broadly expressed in vegetative and reproductive organs, with a particularly high expression in silks (Figure S6). Notably, several genes, such as *ZmKCS2*, *ZmKCS7*, *ZmKCS8*, *ZmKCS14*, and *ZmKCS25*, presented constitutive expression across multiple tissues. Interestingly, certain *ZmKCS* genes presented tissue-specific expression patterns: *ZmKCS20* was uniquely expressed in anthers and pollen; *ZmKCS1*, *ZmKCS10*, and *ZmKCS11* were specifically expressed in silks; *ZmKCS5* was specifically expressed in anthers and roots; and *ZmKCS22* was specifically expressed in stems and leaves. These findings suggest that *ZmKCS* genes may play crucial roles in the development and functionality of specific maize tissues.

**Figure 5:**
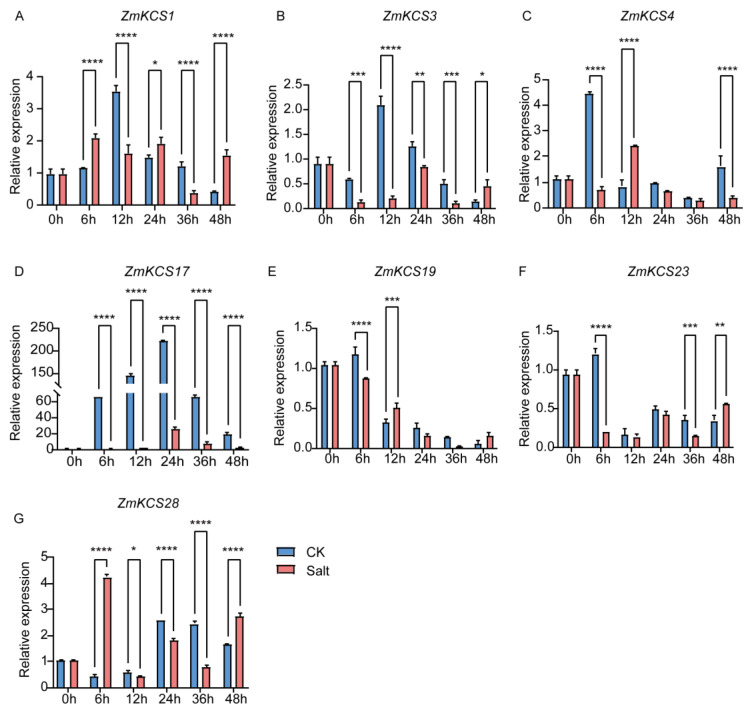
Salt stress response of *ZmKCS* genes. The expression levels of seven *ZmKCS* genes (**A**–**G**) were assessed via qRT-PCR. Maize seedlings were subjected to salt stress (150 mM NaCl), and leaf samples were collected at 0, 6, 12, 24, 36, and 48 h. The data are presented as the means ± standard errors (SEs) of three biological replicates. Statistically significant differences between the control (CK) and salt treatment groups are denoted by asterisks: * *p* < 0.05, ** *p* < 0.01, *** *p* < 0.001, **** *p* < 0.0001 (determined by independent Student’s tests).

**Figure 6:**
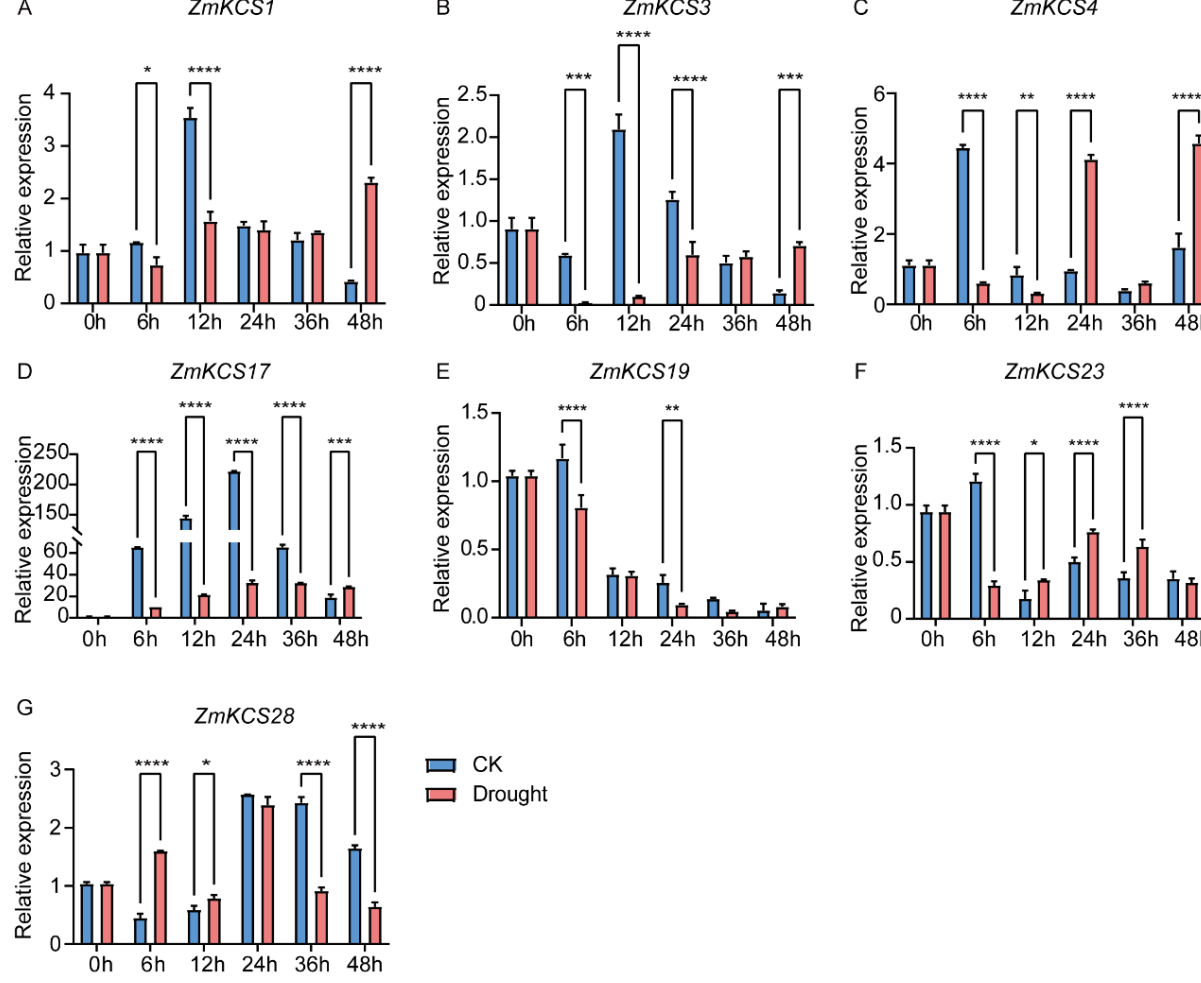
Drought stress response of *ZmKCS* genes. The expression levels of seven *ZmKCS* genes (**A**–**G**) were validated via qRT-PCR. Seedings were subjected to drought (20% PEG6000), and leaves were sampled at 0, 6, 12, 24, 36 and 48 h. Data represent the means ± standard errors (SEs) of three biological replicates. Statistically significant differences between the control (CK) and treatment groups (PEG) are indicated by asterisks (* *p* < 0.05, ** *p* < 0.01, *** *p* < 0.001, **** *p* < 0.0001; independent Student’s *t*-test).

Previous studies have firmly established the significant roles of *KCS* genes in the plant response to abiotic stresses, including drought and salt stresses, and in reactions to plant hormones such as ABA [39]. The identification of *cis*-acting elements related to the abiotic stress response in the promoter regions of *ZmKCS* genes also further suggests their potential functions in various abiotic stress responses. To gain deeper insights into the molecular functions of *KCS* genes in the response of maize to salt and drought stresses, we randomly selected seven genes from distinct subgroups of the phylogenetic tree and examined their expression levels at 0, 6, 12, 24, 36, and 48 h after salt and drought treatments (Figure 5 and Figure 6). The results indicated that the expression levels of these seven *ZmKCS* genes fluctuated, showing either upregulation or downregulation within 48 h after both treatments, with significant expression differences compared with those of the control group observed as early as 6 h after treatment (Figure 5). This rapid response suggests that *ZmKCS* genes are capable of swiftly reacting to abiotic stresses. In response to salt stress, *ZmKCS1* and *ZmKCS28* exhibited similar expression patterns, characterized by an initial increase at 6 h, followed by a subsequent decrease, and a notable upregulation in expression after 48 h of salt treatment. This pattern suggests that these genes may play complex roles in the response to salt stress. In contrast, the expression levels of *ZmKCS3* and *ZmKCS23* were significantly upregulated after 48 h of salt treatment, whereas their expression was downregulated at other time points. Notably, *ZmKCS17* exhibited significant differences in expression compared with the control group at all time points after salt treatment. *ZmKCS4* and *ZmKCS19* presented a noticeable downregulation–upregulation trend between 6 and 12 h of salt treatment. Under drought stress, *ZmKCS1*, *ZmKCS3*, *ZmKCS4*, *ZmKCS17*, and *ZmKCS23* displayed similar expression patterns, with an initial decrease followed by an increase. Conversely, the expression of *ZmKCS28* initially increased but then decreased under drought stress, while that of *ZmKCS19* continued to decrease (Figure 6). Notably, among these differentially expressed genes, *ZmKCS17* presented the most pronounced fold change in expression during both salt and drought treatment, with almost no expression, strongly implying that *ZmKCS17* could be a key gene contributing to the response of maize to abiotic stresses (Figure 5 and Figure 6). In summary, our findings highlight the diversity in expression patterns among various *ZmKCS* genes under salt and drought stress conditions, suggesting that specific *ZmKCS* genes may play distinct roles in the response to abiotic stress, potentially through different mechanisms.

## 3. Discussion

Gene family analysis is crucial for identifying biologically relevant genes and their functions, guiding downstream functional genetic analyses, and elucidating the evolutionary relationships between different species. Understanding the molecular mechanisms that underpin plant growth, development, and responses to environmental stresses is particularly significant [40,41,42]. To date, the *KCS* gene family has been identified across numerous species, revealing its multifaceted roles in development, fertility, and resistance to both biotic and abiotic stresses [4,14,15,16,17,18,19,20,21,22]. The consistent number of *KCS* gene members, approximately 30 in most plants, may reflect the evolutionarily conserved nature of these genes and their crucial biological functions, potentially linked to common ancestral genomic events [43,44,45]. In this study, a comprehensive analysis of the *KCS* gene family in maize yielded valuable insights into its genomic organization, evolutionary history, and potential roles in the stress response. We identified 29 *KCS* genes unevenly distributed across the maize chromosomes, suggesting a complex evolutionary history influenced by gene duplication events. The variation in gene number on each chromosome, ranging from one to eight, and the physical positions of these genes indicate that chromosomal rearrangements and duplications have contributed to the expansion and diversification of the *ZmKCS* genes [42,46].

Evolutionary analysis provides profound insights into the relationships and origins of gene family members during the evolutionary process [47]. Our evolutionary analysis, which includes the construction of a phylogenetic tree and collinearity analysis, revealed a closer relationship between maize and other monocotyledonous plants, particularly sorghum and rice. This underscores the importance of comparative genomics in understanding the evolutionary dynamics of gene families across different species. The structural simplicity and conservation observed within the *ZmKCS* genes, as evidenced by the presence of intronless genes and those with a single intron, suggest a pattern of intron loss or gain during evolution, which may be crucial for the functional integrity of these genes. The identification of conserved motifs, particularly motifs 3 and 5 in ZmKCS proteins, indicates their potential involvement in key biological processes such as fatty acid synthesis and stress response, highlighting the importance of these motifs for protein function.

Lipid metabolism pathways are located mainly in the plastid, endoplasmic reticulum (ER), and peroxisome of plants [48]. In *Arabidopsis*, several *KCS* genes have been reported to be expressed in the ER [49,50,51]. However, our analysis of subcellular localization prediction and experimental verification revealed diverse cellular distributions, with a majority localized at the plasma membrane and dual localization of ZmKCS22 to both the cell membrane and chloroplasts, which is consistent with observations in sorghum [4], highlighting the complexity of protein targeting and function within the cell.

The analysis of *cis*-acting elements in *ZmKCS* gene promoters provides insights into the regulatory mechanisms underlying the response to abiotic stresses and hormone signaling. The presence of various stress-responsive elements suggests that *ZmKCS* genes may play a role in mediating responses to environmental challenges, which is consistent with the role of *KCS* genes reported in multiple species under biological and abiotic stresses [30,31,32,33,34,35]. In addition, gene expression is closely related to gene function [52,53]. The distinct expression patterns of *ZmKCS* genes across various tissues and developmental stages hint at complex regulation critical for maize growth and development. High expression in silks, for example, could indicate a significant role of *ZmKCSs* in the reproductive process, possibly influencing pollination and seed development. This specificity is vital for the reproductive success of maize and could be a target for enhancement in breeding programs focused on improving yield and environmental resilience. The constitutive expression of certain *ZmKCS* genes across multiple tissues implies that they may be involved in fundamental biological processes that are essential for maize growth and development. The expression profiling of *ZmKCS* genes under salt and drought stresses revealed a dynamic response pattern, with both upregulation and downregulation observed, suggesting the potential of these genes to intervene early in abiotic stress responses. Many *ZmKCS* genes respond quickly to salt and drought stresses, indicating functional redundancy among *ZmKCS* members in this process. The functional redundancy in the *ZmKCS* gene family could act as a safeguard against genetic mutations, preserving the stability of key biological processes in maize [54]. On the other hand, the unique functions of individual *ZmKCS* genes might provide specialized responses to particular environmental cues. Understanding the balance between redundancy and uniqueness is crucial for leveraging the full potential of the *ZmKCS* genes in crop improvement and for gaining insights into the evolution of stress response mechanisms in plants. Notably, the pronounced response of *ZmKCS17* to both stresses suggests a central role in mediating maize’s stress response. Further investigation into the molecular pathways regulated by *ZmKCS17* may reveal novel mechanisms of stress tolerance. Moreover, the potential of *ZmKCS17* in genetic engineering is substantial, especially for enhancing stress tolerance in maize. By understanding the regulatory mechanisms and functional roles of *ZmKCS17*, we can devise strategies to modulate their expression or activity, resulting in the creation of maize varieties maize varieties more capable of withstanding environmental challenges. This could have profound implications for agricultural productivity and food security amidst climate change. Together, these findings enhance our understanding of the evolutionary conservation and functional diversity of the *ZmKCS* gene family in maize, highlighting their potential roles in plant adaptation and development.

## 4. Materials and Methods

### 4.1. Identification of KCS Gene Family Members in Maize

To identify *KCS* genes in maize, we downloaded genome files of the maize B73 inbred line from the Ensembl Plants database (http://plants.ensembl.org/index.html (accessed on 10 January 2024)), which included coding sequences (CDSs) and protein sequences. We employed HMMsearch to detect KCS proteins within the maize genome. For this purpose, we utilized domains from the Pfam database (http://pfam.xfam.org/ (accessed on 12 January 2024)), specifically the FAE1/Type III polyketide synthase-like protein (FAE1_CUT1_RppA, Pfam:PF08392) and 3-Oxoacyl-[acyl-carrier-protein (ACP)] synthase III C terminal (ACP_syn_III_C, Pfam:PF08541), to conduct HMM searches across the maize proteome with an e-value threshold of 1.0E-5. The identified KCS protein sequences were then subjected to structural domain confirmation and analysis via online tools, notably the NCBI CD-Search (https://www.ncbi.nlm.nih.gov/cdd/ (accessed on 12 January 2024)) and the SMART database (http://smart.embl-heidelberg.de/ (accessed on 10 January 2024)). Sequences with incorrect or incomplete structural domains were excluded. Ultimately, 29 *ZmKCS* genes were identified and designated on the basis of their chromosomal locations. We subsequently analyzed the amino acid sequences of maize *KCS* family members via the online tool ProtParam (http://web.expasy.org/protparam/ (accessed on 13 January 2024)) to determine their molecular weight (MW), isoelectric point (PI), and amino acid length.

### 4.2. Chromosome Localization Analysis of ZmKCS Genes

We utilized the genomic annotation data from the Ensemble Plant Database to determine the length of the chromosomes and the positional information of the *ZmKCS* genes. MapChart v2.32 software was then used to generate a chromosomal localization map for the *ZmKCS* genes, which were further refined and preserved using Adobe Illustrator CS6 [55].

### 4.3. Phylogenetic Tree Construction and Collinearity Analysis of KCS Genes in Seven Species

To explore the phylogenetic relationships among ZmKCS proteins across different species, a comprehensive phylogenetic tree was constructed via MEGA 7.0 software [56]. This tree included a total of 182 KCS proteins sourced from four monocot plants (maize, sorghum, rice, and *Brachypodium distachyon*) and three dicot plants (*Arabidopsis thaliana*, tomato, and soybean). Branch support was robustly evaluated through 1000 bootstrap iterations. The phylogenetic tree was then refined and visualized using the Evolview online platform [57]. Additionally, we conducted an analysis of duplication events for each *ZmKCS* gene using the Multiple Collinearity Scan Toolkit (MCScanX) with default parameters [58]. By performing BLAST comparisons of all the ZmKCS protein sequences, we identified colinear gene pairs within the maize *KCS* gene family. To further investigate the homology of *KCS* genes between maize and six other monocot or dicot plants, intergenomic collinearity analysis was performed via the TBtools v2.056 software [59].

### 4.4. Analysis of Gene Structure and Protein Conserved Motifs of ZmKCS Genes

The Gene Structure Display Server (GSDS) (http://gsds.cbi.pku.edu.cn (accessed on 10 March 2024)) was used to analyze the exon–intron structure of the *ZmKCS* genes based on their full-length CDS sequences. To predict conserved motifs within the KCS amino acid sequences, the MEME (Motif-based sequence analysis tools) website (https://meme-suite.org/meme/ (accessed on 15 March 2024)) was utilized with specific parameter settings: the optimal motif width ranged from 6 to 50 residues, and the maximum number of motifs was set to 10. The position information of the identified motifs in the ZmKCS protein sequences, stored in XML files, was transformed into graphical representations via the TBtools software [59].

### 4.5. Secondary Structure Prediction and Three-Dimensional Model Construction of ZmKCS Proteins

The Prabi online platform (https://npsa-prabi.ibcp.fr/ (accessed on 18 May 2024)) was utilized to predict the secondary structure of the ZmKCS proteins. A three-dimensional model of each ZmKCS protein was constructed using a homology-based protein modeling approach for a more detailed structural analysis. The amino acid sequences of the ZmKCS proteins were submitted to the SWISS-MODEL website (https://swissmodel.expasy.org/ (accessed on 20 May 2024)) to obtain their three-dimensional structures.

### 4.6. Cis-Acting Element Analysis of ZmKCS Genes Promoter Regions

To investigate the regulatory patterns of gene expression, upstream promoter sequences of 1500 base pairs were extracted from the maize genome at the transcription start site of each *ZmKCS* gene. PlantCARE (*Cis*-acting Regulatory Element, http://bioinformatics.psb.ugent.be/webtools/plantcare/html/ (accessed on 2 June 2024)) was employed to analyze the *cis*-regulatory elements within these promoter regions, providing detailed insights into these elements. Visual representations of these *cis*-regulatory elements were generated through the GSDS online platform.

### 4.7. Subcellular Localisation Analysis of ZmKCS Proteins

The subcellular localization of ZmKCS proteins was predicted via the CELLO website (http://cello.life.nctu.edu.tw/ (accessed on 7 June 2024)). By submitting the protein sequences to the website, we identified potential organelle locations, which were then visualized using TBtools. To validate the bioinformatic prediction, we experimentally verified the localization by randomly selecting four genes from different subgroups based on their predicted localization results. Specific primers, detailed in Table S3, were designed according to the CDSs of selected *ZmKCS* genes, and the accession numbers of selected *ZmKCS* genes were shown in Table S1. The subcellular locations of selected ZmKCSs were determined according to Hu et al., 2023 [60]. The full-length CDS of these genes was amplified using the KOD One^TM^ PCR Master Mix (TOYOBO, Osaka, Japan). Vector linearization was performed using HindIII and KpnI enzymes (NEB, Ipswich, MA, USA). The full-length CDS of the four *ZmKCS* genes was then cloned and inserted into the plant expression vector Super1300: GFP using the ClonExpress^®^II One Step Cloning Kit (Vazyme, Nanjing, China). The constructs containing the GFP-fused proteins were subsequently transformed into Agrobacterium GV3101. These strains were subsequently used to infect the leaves of three-week-old *Nicotiana benthamiana* plants, with three to four tobacco plants infected per gene and two to three leaves injected per plant [61]. On the basis of the predicted subcellular localization, target genes containing PIP2A were co-transformed into tobacco leaves. The tobacco plants were then cultivated in darkness at 28 °C for two days, after which fluorescence images were captured via a Leica TCS SP8 confocal microscopy system (Mannheim, Germany).

### 4.8. Plant Materials, Stress Treatments, and RNA Extraction

Plump B73 seeds were selected, surface sterilized with 75% ethanol for 1 min and then soaked in sterile water for 6 h. The seeds were sown in small pots filled with a mixture of nutrient soil and vermiculite (at a ratio of 3:1) and grown in a greenhouse under a 14 h light/10 h dark photoperiod at 26 °C. When the B73 seedlings reached the three-leaf stage, 150 uniformly growing seedlings were selected and transferred to planting containers, where they were cultured in 1/2 Hoagland nutrient solution to acclimate to hydroponic conditions for two additional days. Each container housed 6 seedlings, each with 1.8 L of 1/2 Hoagland nutrient solution. Following the acclimation, 120 seedlings exhibiting good and consistent growth were selected and divided into two groups—one for salt treatment and the other for drought treatment—For further study, leaf samples were collected immediately post-selection to establish the 0 h baseline. Post-initial sampling, the seedlings were treated with either 150 mM NaCl or 20% polyethylene glycol (PEG) solutions [4,61,62,63,64,65,66], and the timing was initiated. Subsequent leaf samples were collected at 6, 12, 24, 36, and 48 h post-treatment, yielding a total of six time points samples per treatment. For each time point, we collected three biological replicates, with each replicate consisting of a composite sample from three distinct plants. This approach ensures the robustness and reproducibility of our experimental data, enabling a thorough analysis of the maize seedlings’ stress response over time. Additionally, total RNA was extracted from the leaves using the CWBIO Ultrapure RNA Kit (DNase I) (CWBIO, Beijing, China), and its integrity was assessed via 1.2% agarose gel electrophoresis. First-strand cDNA was synthesized using HiScript^®^ III RT SuperMix (+gDNA WIper) (Vazyme, Nanjing, China).

### 4.9. Expression Profiling and qRT-PCR Analysis of ZmKCS Genes

Transcriptome data from a previous study [38] was analyzed to obtain the expression profiles of 25 maize *KCS* genes across various tissues and developmental stages. The normalized gene expression values, represented as Fragments Per Kilobase of Exon per Million Fragments Mapped (FPKM), were log_2_(FPKM + 1) transformed and loaded into TBtools software for further expression analysis. Quantitative real-time polymerase chain reaction (qRT-PCR) was carried out in triplicate for each sample via ChamQ SYBR qPCR Master Mix (Low ROX Premixed) (Vazyme, Nanjing, China) to evaluate the expression levels of 7 randomly selected maize *KCS* genes under salt and drought stresses. The qRT-PCR was conducted using the gene-specific primers listed in Supplementary Table S1 under the following conditions: initial denaturation at 95 °C for 30 s, followed by 40 cycles of 95 °C for 10 s, and 60 °C for 30 s. An ABI 7500 FAST Real-Time PCR System was used for qRT-PCR analysis. The maize Tubulin gene (*Zm00001d013367*) served as a control for sample normalization (Table S2). The relative transcription levels were calculated using the comparative threshold cycle method [67].

## 5. Conclusions

The *ZmKCS* gene family in maize presents a complex pattern of genomic organization, evolutionary relationships, and functional diversity. The structural and functional analysis of these genes provides a foundation for further functional characterization and application in crop improvement strategies aimed at enhancing stress tolerance and yield potential in maize.

## Data Availability

The datasets supporting the results of this article, mainly including the genomic sequences, are available from Gramene website (https://ensembl.gramene.org/Zea_mays/Info/Index (accessed on 10 January 2024)).

## Supplementary Materials

The following supporting information can be downloaded at https://www.mdpi.com/article/10.3390/plants13243507/s1, Figure S1: Localization of *ZmKCS* genes on maize chromosomes; Figure S2: Multiple sequence alignment and domain analysis of AtKCS and ZmKCS proteins; Figure S3: Three-dimensional structural modeling of ZmKCS proteins; Figure S4: Subcellular localization prediction of ZmKCS proteins; Figure S5: Characterization of *cis*-acting elements in the promoter regions of *ZmKCS* genes; Figure S6: Expression profilings of *ZmKCS* genes across various B73 tissues and developmental stages; Table S1: Characterization of the *ZmKCS* genes and ZmKCS proteins; Table S2: The secondary structural statistics of the ZmKCS proteins; Table S3: Primers of full-length CDSs for 4 *ZmKCS* genes; Table S4: Primers of qRT-PCR for *ZmKCS* genes.

## References

[1] Haslam T.M., Kunst L. (2013). Extending the story of very-long-chain fatty acid elongation. Plant Sci..

[2] Bach L., Faure J.D. (2010). Role of very-long-chain fatty acids in plant development, when chain length does matter. Comptes Rendus Biol..

[3] Batsale M., Bahammou D., Fouillen L., Mongrand S., Joubès J., Domergue F. (2021). Biosynthesis and functions of very-long-chain fatty acids in the responses of plants to abiotic and biotic stresses. Cells.

[4] Zhang A., Xu J., Xu X., Wu J., Li P., Wang B., Fang H. (2022). Genome-wide identification and characterization of the KCS gene family in Sorghum (*Sorghum bicolor* (L.) Moench). PeerJ.

[5] Kihara A. (2012). Very long-chain fatty acids: Elongation, physiology and related disorders. J. Biochem..

[6] Erdbrügger P., Fröhlich F. (2020). The role of very long chain fatty acids in yeast physiology and human diseases. Biol. Chem..

[7] Zhukov A.V., Shumskaya M. (2020). Very-long-chain fatty acids (VLCFAs) in plant response to stress. Funct. Plant Biol..

[8] Raffaele S., Leger A., Roby D. (2009). Very long chain fatty acid and lipid signaling in the response of plants to pathogens. Plant Signal Behav..

[9] Schneiter R., Hitomi M., Ivessa A.S., Fasch E.V., Kohlwein S.D., Tartakoff A.M. (1996). A yeast acetyl coenzyme A carboxylase mutant links very-long-chain fatty acid synthesis to the structure and function of the nuclear membrane-pore complex. Mol. Cell Biol..

[10] Paul S., Gable K., Beaudoin F., Cahoon E., Jaworski J., Napier J.A., Dunn T.M. (2006). Members of the *Arabidopsis* FAE1-like 3-ketoacyl-coA synthase gene family substitute for the Elop proteins of saccharomyces cerevisiae. J. Biol. Chem..

[11] De Bigault Du Granrut A., Cacas J.L. (2016). How very-long-chain fatty acids could signal stressful conditions in plants?. Front. Plant Sci..

[12] Hu W., Chen L., Qiu X., Wei J., Lu H., Sun G., Ma X., Yang Z., Zhu C., Hou Y. (2020). *AKR2A* participates in the regulation of cotton fiber development by modulating biosynthesis of very-long-chain fatty acids. Plant Biotechnol. J..

[13] Bernard A., Joubès J. (2013). *Arabidopsis* cuticular waxes: Advances in synthesis, export and regulation. Prog. Lipid Res..

[14] Xing J., Zhu M., Xu H., Liu H., Wang Y. (2023). Genome-wide characterization of the role of the *KCS* gene family in *Allium fistulosum* L. as regulators of abiotic stress responses. Sci. Hortic..

[15] Gong Y., Wang D., Xie H., Zhao Z., Chen Y., Zhang D., Jiao Y., Shi M., Lv P., Sha Q. (2023). Genome-wide identification and expression analysis of the KCS gene family in soybean (*Glycine max*) reveal their potential roles in response to abiotic stress. Front. Plant Sci..

[16] Khan U.M., Rana I.A., Shaheen N., Raza Q., Rehman H.M., Maqbool R., Khan I.A., Atif R.M. (2023). Comparative phylogenomic insights of KCS and ELO gene families in *Brassica* species indicate their role in seed development and stress responsiveness. Sci. Rep..

[17] Tong T., Fang Y., Zhang Z., Zheng J., Zhang X., Li J., Niu C., Xue D., Zhang X. (2021). Genome-wide identification and expression pattern analysis of the KCS gene family in barley. Plant Growth Regul..

[18] Rizwan H.M., Shaozhong F., Li X., Bilal Arshad M., Yousef A.F., Chenglong Y., Shi M., Jaber M.Y.M., Anwar M., Hu S.-Y. (2022). Genome-wide identification and expression profiling of KCS gene family in passion fruit (*Passiflora edulis*) under *Fusarium kyushuense* and drought stress conditions. Front. Plant Sci..

[19] Wang H., He T., Huang C., Wang K., Shi D., Si X., Xu Y., Lyu S., Huang J., Li Y. (2023). Genome-wide identification of *KCS* gene family in *Carya illinoinensis* and their roles under abiotic stress conditions. Sci. Hortic..

[20] Liu X., Zhao Z., Yang Y., Xu H., Bi Q., Wang L. (2023). Genome-wide identification and expression analysis of the KCS gene family in yellow horn reveal their putative function on abiotic stress responses and wax accumulation. Horticulturae.

[21] Huai D., Xue X., Li Y., Wang P., Li J., Yan L., Chen Y., Wang X., Liu N., Kang Y. (2020). Genome-wide identification of peanut KCS genes reveals that *AhKCS1* and *AhKCS28* are involved in regulating VLCFA contents in seeds. Front. Plant Sci..

[22] Zhang J., Zhang C., Li X., Liu Z.-Y., Liu X., Wang C.L. (2023). Comprehensive analysis of KCS gene family in pear reveals the involvement of *PbrKCSs* in cuticular wax and suberin synthesis and pear fruit skin formation. Plant Mol. Biol..

[23] Todd J., Post-Beittenmiller D., Jaworski J.G. (1999). *KCS1* encodes a fatty acid elongase 3-ketoacyl-CoA synthase affecting wax biosynthesis in *Arabidopsis thaliana*. Plant J..

[24] Luo N., Wang Y., Liu Y., Wang Y., Guo Y., Chen C., Gan Q., Song Y., Fan Y., Jin S. (2024). 3-ketoacyl-CoA synthase 19 contributes to the biosynthesis of seed lipids and cuticular wax in *Arabidopsis* and abiotic stress tolerance. Plant Cell Environ..

[25] Huang H., Yang X., Zheng M., Lü S., Zhao H. (2023). Fine-tuning the activities of β-KETOACYL-COA SYNTHASE 3 (KCS3) and KCS12 in *Arabidopsis* Is essential for maintaining cuticle integrity. J. Exp. Bot..

[26] Yang S., Tang H., Wei X., Zhao Y., Wang Z., Su H., Niu L., Yuan Y., Zhang X. (2022). *BrWAX3*, encoding a β-ketoacyl-CoA synthase, plays an essential role in cuticular wax biosynthesis in chinese cabbage. Int. J. Mol. Sci..

[27] Kim J., Lee S.B., Suh M.C. (2021). *Arabidopsis* 3-ketoacyl-CoA synthase 4 is essential for root and pollen tube growth. J. Plant Biol..

[28] Chai M., Queralta Castillo I., Sonntag A., Wang S., Zhao Z., Liu W., Du J., Xie H., Liao F., Yun J. (2021). A seed coat-specific β-ketoacyl-CoA synthase, *KCS12*, is critical for preserving seed physical dormancy. Plant Physiol..

[29] Yang T., Li Y., Liu Y., He L., Liu A., Wen J., Mysore K.S., Tadege M., Chen J. (2021). The 3-ketoacyl-CoA synthase WFL is involved in lateral organ development and cuticular wax synthesis in *Medicago truncatula*. Plant Mol. Biol..

[30] Shaheenuzzamn M., Shi S., Sohail K., Wu H., Liu T., An P., Wang Z., Hasanuzzaman M. (2021). Regulation of cuticular wax biosynthesis in plants under abiotic stress. Plant Biotechnol. Rep..

[31] Liu B., Sun Y., Li X., Guo D., Zhao L., Ma C., Wang L., Wang S. (2023). β-ketoacyl-CoA synthase improves the drought tolerance of root restricted grown grapevines by regulating the cuticular wax biosynthesis. Sci. Hortic..

[32] Wang Y., Liu Y., Pan X., Wan Y., Li Z., Xie Z., Hu T., Yang P. (2023). A 3-ketoacyl-CoA synthase 10 (*KCS10*) homologue from *Alfalfa* enhances drought tolerance by regulating cuticular wax biosynthesis. J. Agric. Food Chem..

[33] Tariq F., Zhao S., Ahmad N., Wang P., Shao Q., Ma C., Yang X. (2022). Overexpression of β-ketoacyl CoA synthase 2B.1 from *Chenopodium quinoa* promotes suberin monomers’ production and salt tolerance in *Arabidopsis thaliana*. Int. J. Mol. Sci..

[34] Wang Q., Du X., Zhou Y., Xie L., Bie S., Tu L., Zhang N., Yang X., Xiao S., Zhang X. (2020). The β-ketoacyl-CoA synthase *KCS13* regulates the cold response in cotton by modulating lipid and oxylipin biosynthesis. J. Exp. Bot..

[35] Majid M.U., Ashraf R., Jabbar B., Arif U., Batool F., Hassan S., Rashid B. (2024). Overexpression of *Gossypium arboreum* 3-ketoacyl-CoA synthase 6 (*GaKCS6*) gene enhanced leaf epicuticle wax in *Gossypium hirsutum* L. and improved tolerance against whitefly. Biocatal. Agric. Biotechnol..

[36] Ranum P., Peña-Rosas J.P., Garcia-Casal M.N. (2014). Global maize production, utilization, and consumption. Ann. N.Y. Acad. Sci..

[37] Salika R., Riffat J. (2021). Abiotic stress responses in maize: A review. Acta Physiol. Plant.

[38] Chen J., Zeng B., Zhang M., Xie S., Wang G., Hauck A., Lai J. (2014). Dynamic transcriptome landscape of maize embryo and endosperm development. Plant Physiol..

[39] Macková J., Vašková M., Macek P., Hronková M., Schreiber L., Šantrůček J. (2013). Plant response to drought stress simulated by ABA application: Changes in chemical composition of cuticular waxes. Environ. Exp. Bot..

[40] Wu Q., Chen Y., Zou W., Pan Y.-B., Lin P., Xu L., Grisham M.P., Ding Q., Su Y., Que Y. (2023). Genome-wide characterization of sugarcane catalase gene family identifies a ScCAT1 gene associated disease resistance. Int. J. Biol. Macromol..

[41] Zhu J., Li W., Zhou Y., Pei L., Liu J., Xia X., Che R., Li H. (2021). Molecular characterization, expression and functional analysis of acyl-CoA-binding protein gene family in maize (*Zea mays*). BMC Plant Biol..

[42] Fang H., Shan T., Gu H., Chen J., Qi Y., Li Y., Saeed M., Yuan J., Li P., Wang B. (2024). Identification and characterization of ACR gene family in maize for salt stress tolerance. Front. Plant Sci..

[43] Fernández R., Gabaldón T. (2020). Gene gain and loss across the metazoan tree of life. Nat. Ecol. Evol..

[44] Bowles A.M.C., Bechtold U., Paps J. (2020). The origin of land plants is rooted in two bursts of genomic novelty. Curr. Biol..

[45] Domazet-Lošo M., Široki T., Šimičević K., Domazet-Lošo T. (2024). Macroevolutionary dynamics of gene family gain and loss along multicellular eukaryotic lineages. Nat. Commun..

[46] Feng W., Mehari T.G., Fang H., Ji M., Qu Z., Jia M., Wang D., Ditta A., Khan M.K.R., Cao Y. (2023). Genome-wide identification of the geranylgeranyl pyrophosphate synthase (GGPS) gene family involved in chlorophyll synthesis in cotton. BMC Genom..

[47] Li S., Wei L., Gao Q., Xu M., Wang Y., Lin Z., Holford P., Chen Z.H., Zhang L. (2023). Molecular and phylogenetic evidence of parallel expansion of anion channels in plants. Plant Physiol..

[48] Li H., Peng Z., Yang X., Wang W., Fu J., Wang J., Han Y., Chai Y., Guo T., Yang N. (2013). Genome-wide association study dissects the genetic architecture of oil biosynthesis in maize kernels. Nat. Genet..

[49] Beaudoin F., Wu X., Li F., Haslam R.P., Markham J.E., Zheng H., Napier J.A., Kunst L. (2009). Functional characterization of the *Arabidopsis* beta-ketoacyl-coenzyme A reductase candidates of the fatty acid elongase. Plant Physiol..

[50] Kim J., Jung J.H., Lee S.B., Go Y.S., Kim H.J., Cahoon R., Markham J.E., Cahoon E.B., Suh M.C. (2013). *Arabidopsis* 3-ketoacyl-Coenzyme A synthase9 is involved in the synthesis of tetracosanoic acids as precursors of cuticular waxes, suberins, sphingolipids, and phospholipids. Plant Physiol..

[51] Pascal S., Bernard A., Deslous P., Gronnier J., Fournier-Goss A., Domergue F., Rowland O., Joubès J. (2019). *Arabidopsis* CER1-LIKE1 functions in a cuticular very-long-chain alkane-forming complex. Plant Physiol..

[52] Srivastava A.K., Lu Y., Zinta G., Lang Z., Zhu J.-K. (2018). UTR-dependent control of gene expression in plants. Trends Plant Sci..

[53] Cavalli G., Heard E. (2019). Advances in epigenetics link genetics to the environment and disease. Nature.

[54] Pérez-Pérez J.M., Esteve-Bruna D., Gonzalez-Bayon R., Caldana C., Hannah M.A., Willmitzer L., Ponce M.R., Micol J.L. (2013). Functional redundancy and divergence within the Arabidopsis RETICULATA-RELATED gene family. Plant Physiol..

[55] Voorrips R.E. (2002). MapChart: Software for the graphical presentation of linkage maps and QTLs. J. Hered..

[56] Kumar S., Stecher G., Tamura K. (2016). MEGA7: Molecular evolutionary genetics analysis version 7.0 for bigger datasets. Mol. Biol. Evol..

[57] He Z., Zhang H., Gao S., Lercher M.J., Chen W.H., Hu S. (2016). Evolview v2: An online visualization and management tool for customized and annotated phylogenetic trees. Nucleic Acids Res..

[58] Wang Y., Tang H., DeBarry J.D., Tan X., Li J., Wang X., Lee T.-h., Jin H., Marler B., Guo H. (2012). MCScanX: A toolkit for detection and evolutionary analysis of gene synteny and collinearity. Nucleic Acids Res..

[59] Chen C., Chen H., Zhang Y., Thomas H.R., Frank M.H., He Y., Xia R. (2020). TBtools: An integrative toolkit developed for interactive analyses of big biological data. Mol. Plant.

[60] Hu K., Dai Q., Ajayo B.S., Wang H., Hu Y., Li T., Huang H., Liu H., Liu Y., Wang Y. (2023). Insights into *ZmWAKL* in maize kernel development: Genome-wide investigation and GA-mediated transcription. BMC Genom..

[61] Fang H., Fu X., Ge H., Jia M., Ji J., Zhao Y., Qu Z., Cui Z., Zhang A., Wang Y. (2024). Genetic analysis and candidate gene identification of salt tolerance-related traits in maize. J. Integr. Agric..

[62] Zhao H., Li Z., Wang Y., Wang J., Xiao M., Liu H., Quan R., Zhang H., Huang R., Zhu L. (2022). Cellulose synthase-like protein *OsCSLD4* plays an important role in the response of rice to salt stress by mediating abscisic acid biosynthesis to regulate osmotic stress tolerance. Plant Biotechnol. J..

[63] Liu P., Zhang Y., Zou C., Yang C., Pan G., Ma L., Shen Y. (2022). Integrated analysis of long non-coding RNAs and mRNAs reveals the regulatory network of maize seedling root responding to salt stress. BMC Genom..

[64] Shivakrishna P., Reddy K.A., Rao D.M. (2018). Effect of PEG-6000 imposed drought stress on RNA content, relative water content (RWC), and chlorophyll content in peanut leaves and roots. Saudi J. Biol. Sci..

[65] Altaf A., Gull S., Zhu X., Zhu M., Rasool G., Ibrahim M.E.H., Aleem M., Uddin S., Saeed A., Shah A.Z. (2021). Study of the effect of peg-6000 imposed drought stress on wheat (*Triticum aestivum* L.) cultivars using relative water content (RWC) and proline content analysis. Pak. J. Agric. Sci..

[66] Qi Y., Ma L., Ghani M.I., Peng Q., Fan R., Hu X., Chen X. (2023). Effects of drought stress induced by hypertonic polyethylene glycol (PEG-6000) on Passiflora edulis sims physiological properties. Plants.

[67] Livak K.J., Schmittgen T.D. (2001). Analysis of relative gene expression data using real-time quantitative PCR and the 2^−ΔΔCT^ method. Methods.

