# Supplementary material for: A Comprehensive Analysis In Silico of KCS Genes in Maize Revealed Their Potential Role in Response to Abiotic Stress
Source: Plants (Basel). 2024 Dec 16;13(24):3507. doi: 10.3390/plants13243507 (PMC11676716; doi:10.3390/plants13243507)
Supplement: Supplementary file 1 [file plants-13-03507-s001.zip › Supplementary figures.pdf]

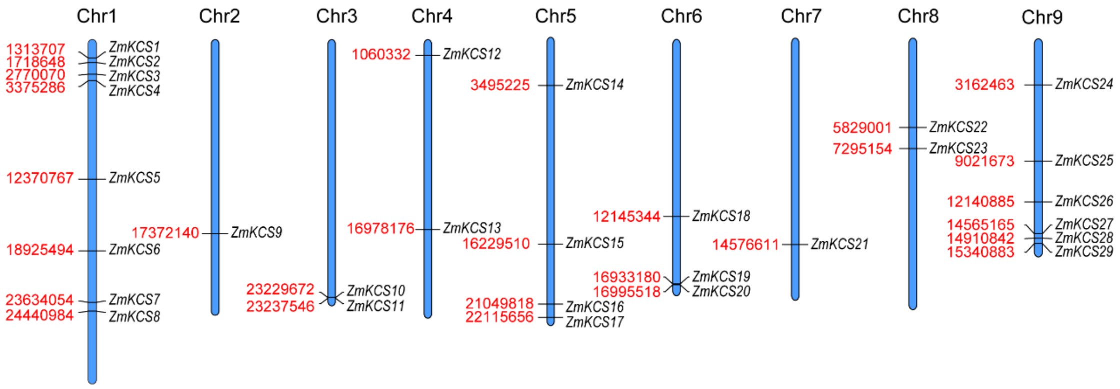

Figure S1. Localization of *ZmKCS* genes on maize chromosomes. Vertical lines represent chromosomes, labeled with numbers at the top. Red numbers on the left indicate the physical positions of *ZmKCS* genes (in basepair, bp), with gene ID near their locations on the right.

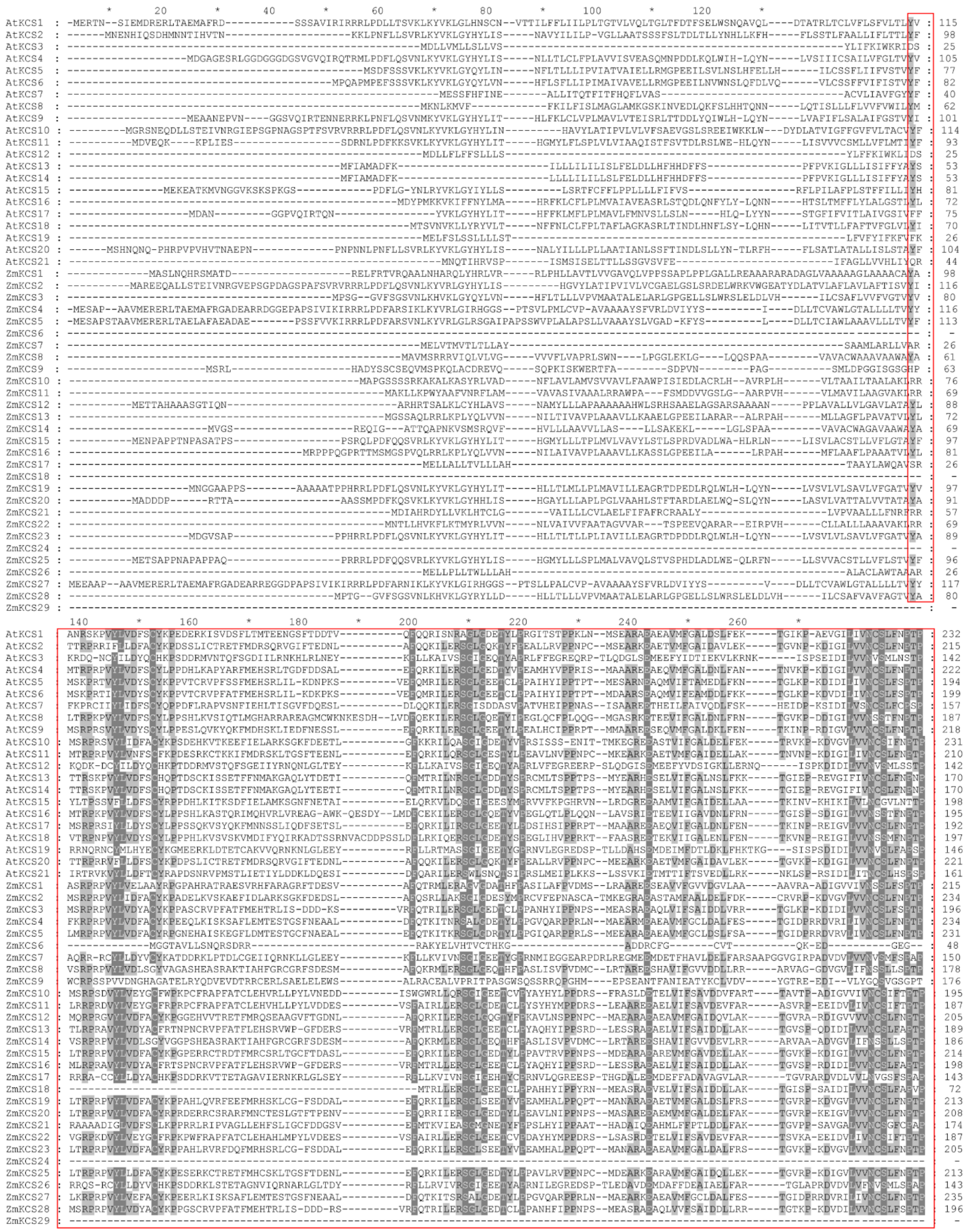

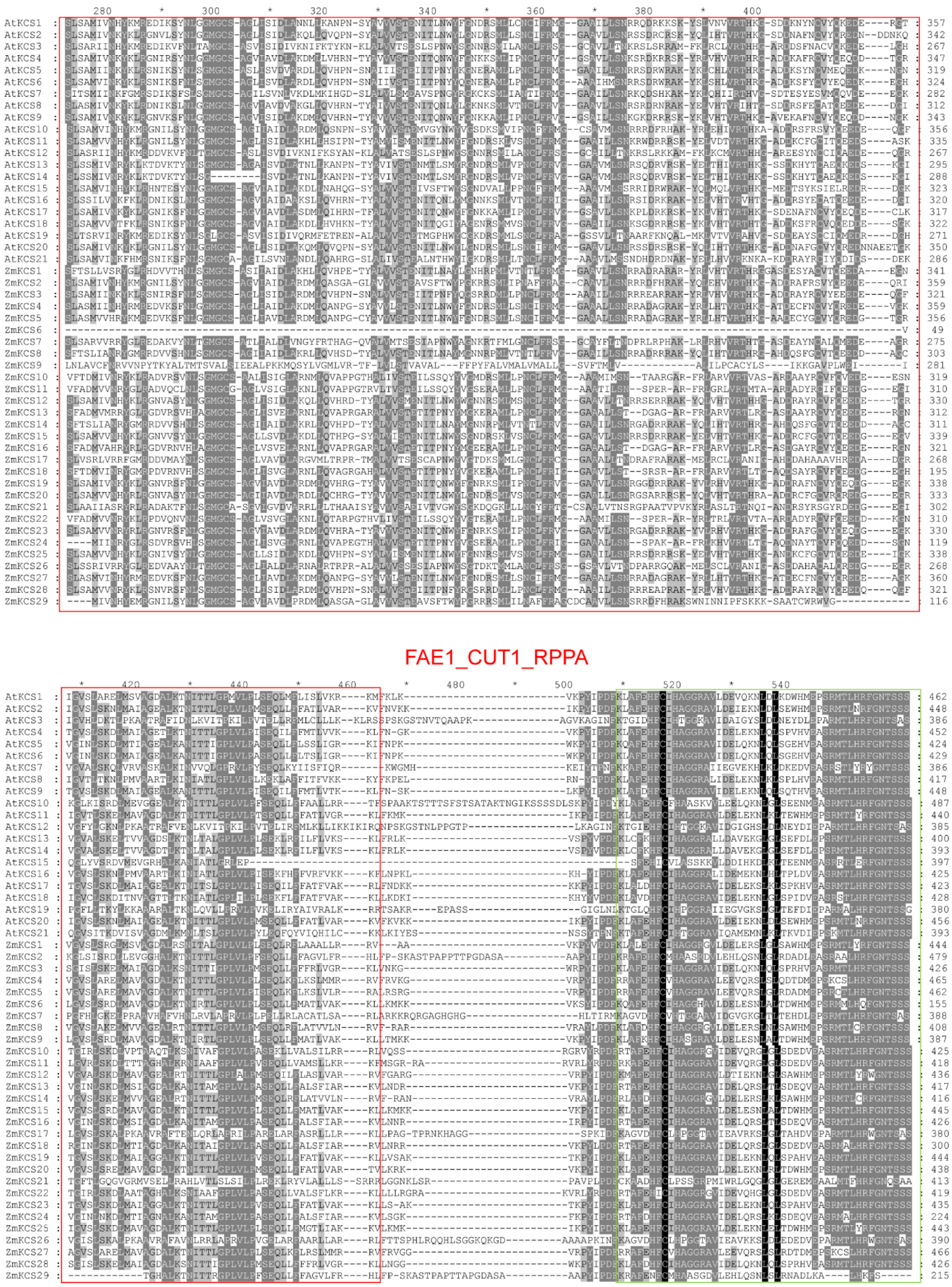
FAE1_CUT1_RPPA

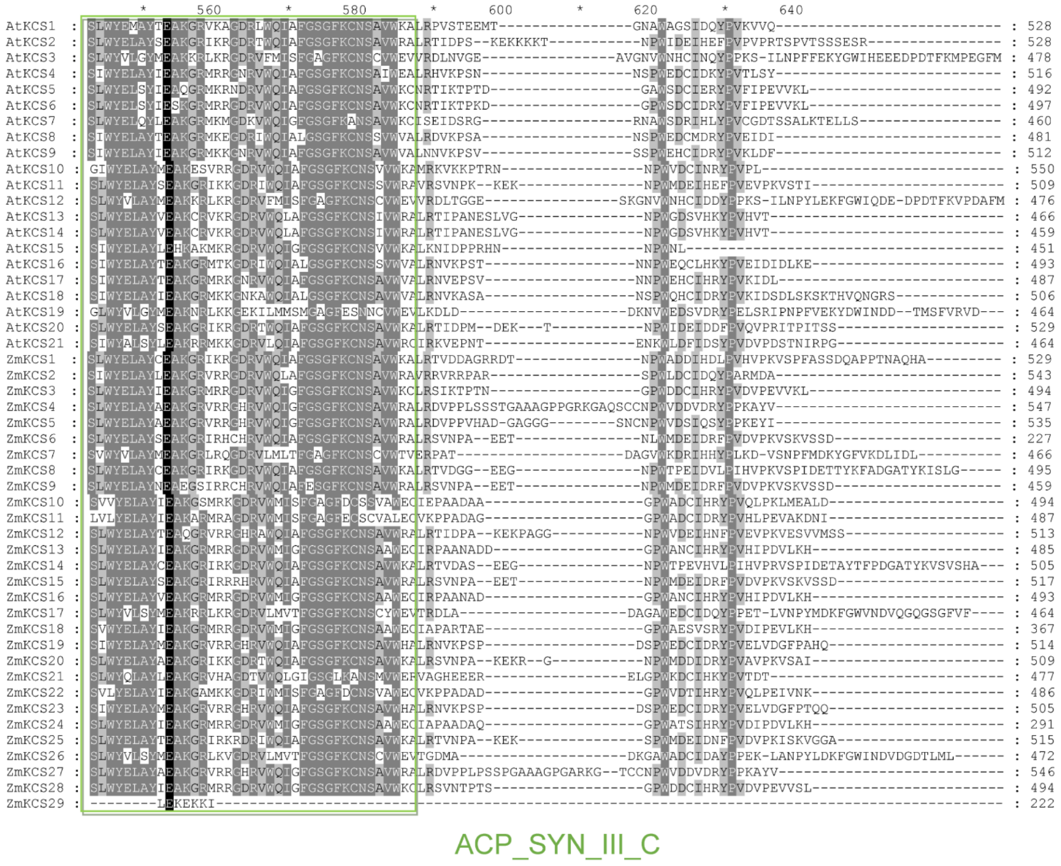

Figure S2. Multiple sequence alignment and domain analysis of AtKCS and ZmKCS proteins.

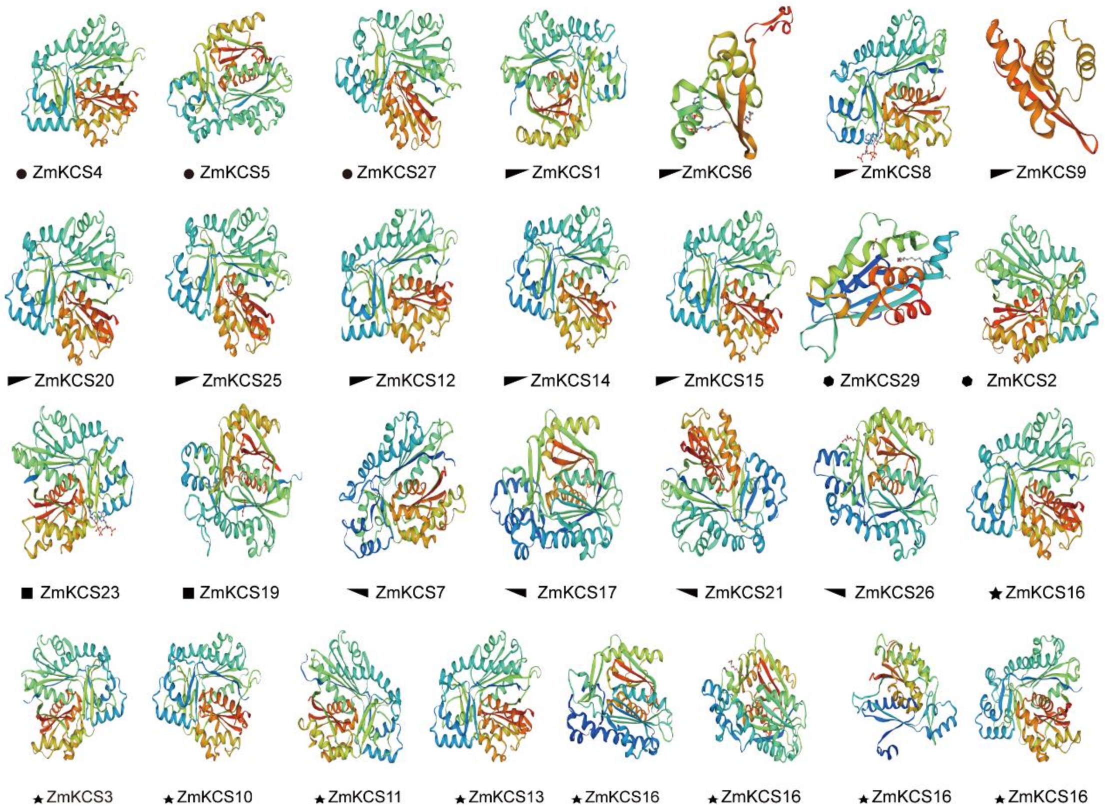

Figure S3 Three-dimensional structural modeling of ZmKCS proteins. Utilizing homology modeling techniques, the ZmKCS proteins were structurally analyzed and modeled via the SWISS-MODEL platform. Each 3D model is presented against a white background, with proteins from distinct subgroups denoted by unique symbols at the base of each model: ● represents Group 1; ◤ represents Group 2; ⬣ represents Group 3; ■ represents Group 4; ◥ represents Group 5; and ★ represents Group 6, facilitating the visual differentiation of subfamily-specific structural features.

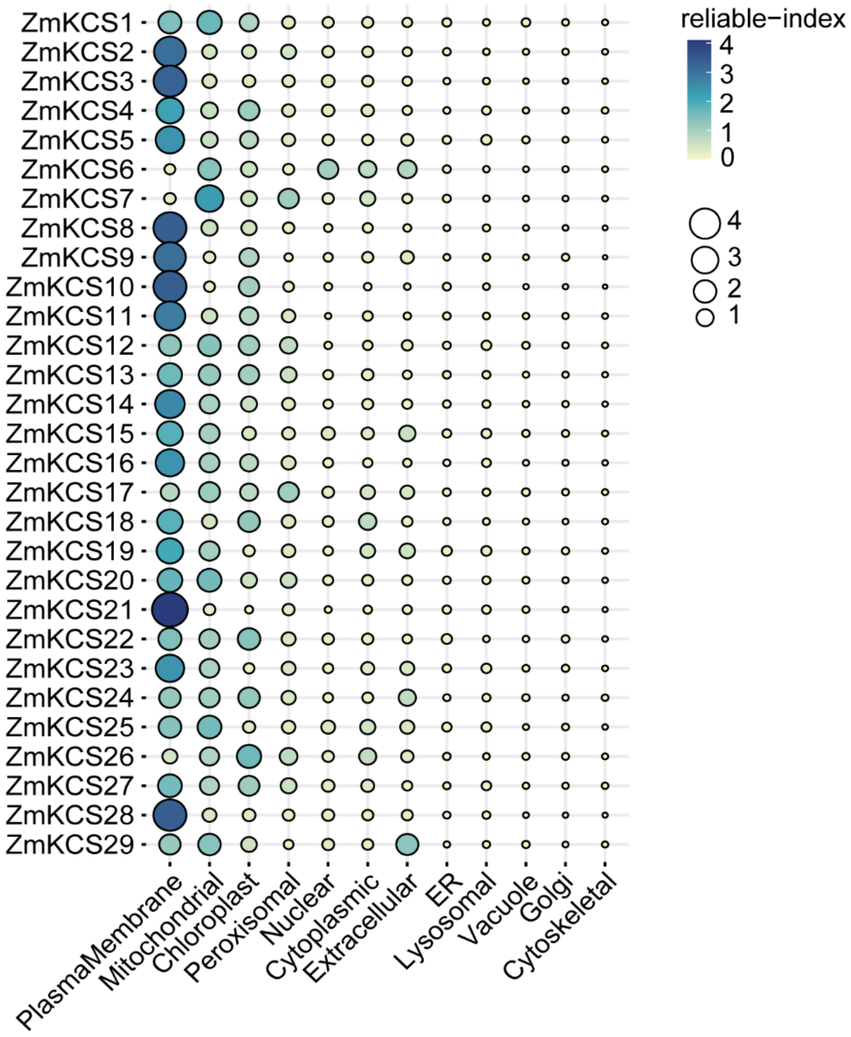

Figure S4 Subcellular localization prediction of ZmKCS proteins. The varying colors and size of the circles denote the confidence levels associated with the prediction outcomes. Displayed on the left are the identifiers for each ZmKCS protein, while the anticipated subcellular localizations for these proteins are delineated at the bottom.

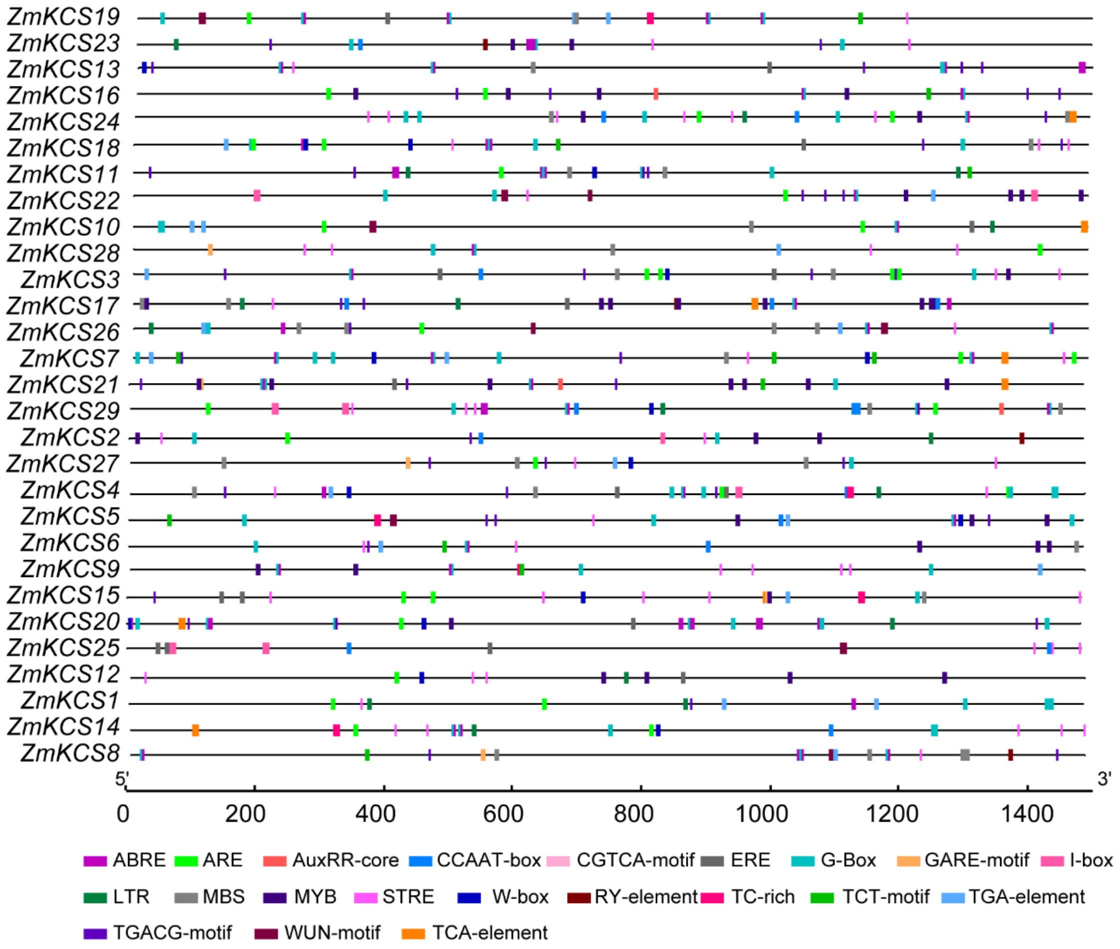

Figure S5. Characterization of *cis*-acting elements in the promoter regions of *ZmKCS* genes. Various *cis*-acting elements are distinguished by unique color coding. The names of the genes are presented on the left side of the figure. The lengths of the promoter sequences are accurately depicted to scale, facilitating a comprehensive comparison across different genes.

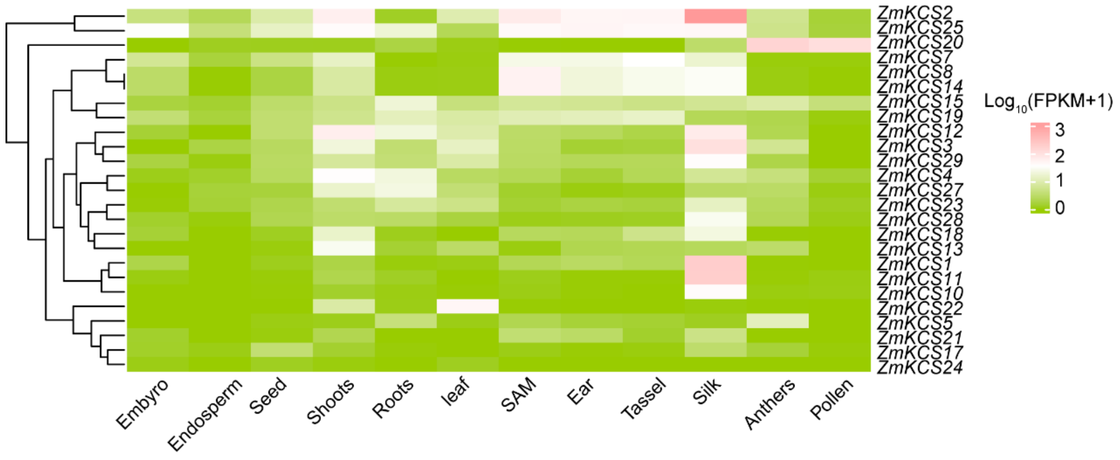

Figure S6. Expression profilings of *ZmKCS* genes across various B73 tissues and developmental stages.
